# Disparities in obesity among rural and urban residents in a health disparate region

**DOI:** 10.1186/1471-2458-14-1051

**Published:** 2014-10-08

**Authors:** Jennie L Hill, Wen You, Jamie M Zoellner

**Affiliations:** Department of Human Nutrition, Foods and Exercise, Virginia Tech, Blacksburg, Virginia USA; Department of Agriculture and Applied Economics, Virginia Tech, Blacksburg, Virginia USA

**Keywords:** Community-based participatory research, Physical activity, Fruit and vegetable intake, Health disparities

## Abstract

**Background:**

The burden of obesity and obesity-related conditions is not borne equally and disparities in prevalence are well documented for low-income, minority and rural adults in the United States. The current literature on rural versus urban disparities is largely derived from national surveillance data which may not reflect regional nuances. There is little practical research that supports the reality of local service providers such as county health departments that may serve both urban and rural residents in a given area. Conducted through a community-academic partnership, the primary aim of this study is to quantify the current levels of obesity (BMI), fruit and vegetable (FV) intake and physical activity (PA) in a predominately rural health disparate region. Secondary aims are to determine if a gradient exists within the region in which rural residents have poorer outcomes on these indicators compared to urban residents.

**Methods:**

Conducted as part of a larger ongoing community-based participatory research (CBPR) initiative, data were gathered through a random digit dial telephone survey using previously validated measures (n = 784). Linear, logistic and quantile regression models are used to determine if residency (i.e. rural, urban) predicts outcomes of FV intake, PA and BMI.

**Results:**

The majority (72%) of respondents were overweight (BMI = 29 ± 6 kg/m^2^), with 29% being obese. Only 9% of residents met recommendations for FV intake and 38% met recommendations for PA. Statistically significant gradients between urban and rural and race exist at the upper end of the BMI distribution. In other words, the severity of obesity is worse among black compared to white and for urban residents compared to rural residents.

**Conclusions:**

These results will be used by the community-academic partnership to guide the development of culturally relevant and sustainable interventions to increase PA, increase FV intake and reduce obesity within this health disparate region. In particular, local stakeholders may wish to address disparities in BMI by allocating resources to the vulnerable groups identified.

## Background

Prevalence of obesity among adults is epidemic in the United States [1–3]. The burden of obesity and obesity-related conditions is not borne equally as low-income and minority populations suffer higher prevalence of obesity and increased co-morbid conditions [4–9]. Additionally, disparities in obesity and obesity-related conditions are evident by geographic location. Notably, the southern states in the U.S. have higher burdens of obesity than northern states. Nation-wide, rural populations exhibit higher prevalence of obesity and obesity-related outcomes such as type 2 diabetes [10–12]. Rates of mortality and morbidity from chronic health conditions are higher among rural populations when compared to their urban or suburban counterparts [10, 11, 13].

From social determinants of health framework, increased prevalence of obesity in rural populations stems in part, from *‘downstream’* behavioral factors such as physical inactivity and poor diet among rural populations [11, 12, 14]. However, these behavioral patterns are influenced by *‘upstream’* determinants such as lower educational attainment and lower SES also characterizing many rural areas [15]. Additional structural constraints in rural regions including lack of medical providers and increased distances to obtain medical care act to perpetuate disparities in health outcomes [9, 16]. Additionally, rural residents may lack access to primary prevention efforts and reduced access to the facilities or amenities that foster healthy behaviors (i.e. recreations centers, grocery stores) [17–19]. Understanding the causes of these disparities is essential to the development and implementation of effective interventions to address obesity and its related conditions in rural regions [5].

The delivery of prevention services in rural areas is further hindered by the logistics of serving hard-to-reach populations dispersed over large geographic areas [17, 19]. The current literature exploring disparities by rural or urban residency is typically based on national or state level surveillance data. While these data provide important information to understand disparities, they do not necessarily reflect the reality of service providers such as county health departments or private healthcare systems that may serve both urban and rural residents in a given region. Further, small- to mid-sized cities are certainly ‘urban’ compared to rural areas, but they may not have the same population density or access to resources that is typical of urban or suburban population centers. There is little practical research that considers these types of regions. Yet there is a need to develop health promotion strategies that address the resources and delivery systems in these regions to maximize sustainability of programs.

Community-based participatory research (CBPR) is one method by which researchers can reach vulnerable populations and leverage local expertise to aid in the identification of the problem and solutions [20–22]. The Dan River Partnership for a Healthy Community (DRPHC) is a community-academic partnership operating under CBPR principles in the health disparate Dan River Region (DRR) [23, 24]. To support community-created causal models for obesity [23] and initiatives by the DRPHC, the need for locally generated surveillance data on health outcomes and behavioral factors related to obesity was identified as a priority. Importantly, the overall goal was to provide baseline data for the DRPHC to evaluate the success of obesity-related initiatives and to effectively identify target populations for programming aimed at reducing obesity in the region. Therefore, the primary aim of the current study is to quantify the current levels of obesity and the related behavioral determinants of fruit and vegetable intake (FV) and physical activity (PA) in the region. Secondary aims are to determine if differences in these health behaviors and outcomes exist based on socio-demographic characteristics and to determine if a gradient exists within the region in which rural residents have poorer health outcomes compared to those who live in town. As compared to urban residents, we hypothesized those residing in the outlying rural counties as well as those with lower educational attainment and minority groups would demonstrate higher BMIs, lower rates of PA and lower FV intake.

## Methods

All study activities were approved by Virginia Tech IRB and survey participants provided verbal informed consent prior to completing study activities.

### Study area

The DRR, an educationally and economically disadvantaged region in south central Virginia and north central North Carolina, is federally designated as a medically under-served area/population (MUA/MUP) [25–27]. Geographically the region includes 3 counties covering approximately 1,800 square miles (mi^2^) with a total population across counties of 137,000 [28]. Using the USDA Rural Urban Community Area Codes (RUCAs), all census tracts in the 3 counties are classified as rural with RUCA codes >4 [29]. Population density in the Virginia counties is 65.5 and 141.6 persons/ mi^2^ and in the North Carolina county 55.8 persons/ mi^2^ while the state-wide population density is 202.0 and 191.1 persons/mi^2^ for Virginia and North Carolina [28]. This predominately rural area is anchored by a mid-size regional city (approximately 43,000 residents) covering 44 mi^2^ and another nearby town of approximately 10,000 residents. Resources for the region, including healthcare, retail, institutions of higher education, and large employers are located within the regional city. Thus, while the DRR is rural region, community partners largely recognize those residents living within the regional city and town have access to resources that may be dramatically different than those in the outlying county. For the purposes of this study, we define ‘urban’ residents as those who live within the city limits of the aforementioned city and town. Residents who live outside the city limits are classified as ‘rural’. Based on those urban and rural classifications, the rural population for our study area is 70% white and 26% black with a mean household income of $46,986 and 9% of the rural population has a 4-year college degree. While the urban population is about 49% white and 47% black; with a mean household income of $43,316 and 11% with a 4-year college degree [28].

### Data collection

#### Sampling

A professional survey unit was contracted to create sampling frames and conduct the telephone survey. A random proportional sampling frame was created based on the population for each of the aforementioned 3 counties and the 2 cities, including listed and unlisted land lines and cell phone numbers. Working in collaboration with community partners, the DRPHC initiated a regional media blitz approximately 2 weeks prior to the survey unit initiating phone calls to enhance participation in the telephone survey. Informational emails and flyers were distributed through DRPHC member list serves for printing and circulation. Additional print and audio announcements were distributed through local media including newspapers, radio shows and television programming. These announcements provided information on the DRPHC, the purpose of the telephone survey and encouraged residents to answer and complete the survey if contacted by the survey unit. The survey took approximately 25 minutes and all participants in the random and non-random sample received a $20 gift card for completion of the telephone survey.

### Survey development and testing

Modeled after the Virginia and National Behavioral Risk Factor Surveillance System (BRFSS) surveys for 2011 [30], the telephone survey was comprised of ten modules. The modules reported here include socio-demographics, physical activity, fruit and vegetable intake and BMI. The survey unit conducted a pre-test within the region (n = 22), resulting in minor adaptions to wording and detailed instructions and clarifications for the survey unit. Results from the pilot test were not significantly different than the full sample therefore the pilot respondents are included for analyses.

### Outcome measures

The valid and reliable Godin-Shephard leisure time exercise questionnaire [31] measured minutes of PA and it was scored according to published protocols. Physical activity is reported as minutes/week of physical activity and meeting recommendations is defined using the 2008 Physical Activity Guidelines for adults as >150 minutes of moderate-vigorous activity and 2 days strength training [32]. The valid and reliable National Cancer Institute Fruit and Vegetable screener measured FV intake [33]. This short screener asks participants to report on the frequency and portion size for nine different food items, and is appropriate for population based surveillance and telephone surveys^31^. Both fruit and vegetable intake (FV) is reported as mean servings/day and meeting recommendations is defined as >5cups/day. Using the established kg/m^2^ formula, BMI was calculated from self-reported height and weight. Socio-demographic variables including age, gender, race, income, education and employment status were also collected. Categorical socio-demographics were collapsed to eliminate empty cells.

### Statistical analyses

Descriptive statistics including frequencies, means, and standard deviations were computed for the covariates, independent and dependent variables using SPSS 20.0. Using Stata 12.1, linear, logistic and quantile regression models are used to determine if residency (i.e. rural, urban or housing) predicts continuous or dichotomized outcomes of FV intake, PA and BMI. To achieve unbiased and consistent residency effects, these models control for individual level covariates including gender, race, education level and employment status. Quantile regressions were used to explore the potential heterogeneous residency effects along the outcomes’ distributions.

## Results

Our sample consists of 784 completed surveys (77% response rate). Characteristics of the study sample are presented in Table 1. For the total sample, the mean age was 56(±15.3) years. Seventy-three percent of respondents were female, 78% white, and 30% reported income of < $20,000. There are differences in socio-demographic characteristics by rural or urban residency for race, income, marital status and employment (Table 1). Table 2 summarizes the means and standard deviations for the primary outcomes explored in this study-FV intake, PA, and BMI.

**Table 1:** **Characteristics of study sample by rural and urban residency**

Characteristic	Total sample	Urban	Rural	p-value*
	N=784	n=210	n=574	
Age, M ± SD	56.4±15.3	61.6±14.7	59.1± 15.6	0.14
Gender	%(n)	%(n)	%(n)	
Female	73 (573)	74 (156)	73 (417)	0.36
Race				<.001
White	76(578)	68(137)	78(441)	
Black	22(167)	29(59)	19(108)	
More than 1 race	2(21)	3(7)	3(14)	
Income				0.004
<$20,000	34(221)	44(75)	30(146)	
$20,000-$50,000	39(257)	32(54)	42(203)	
>$50,000	27(178)	24(42)	28(136)	
Education				0.65
< HS	15(119)	14(30)	16(89)	
HS diploma/GED	33(224)	35(72)	35(202)	
Some college	31(245)	30(63)	31(182)	
College grad or higher	21(145)	21(45)	18(100)	
Employment				0.01
Employed	36(277)	34(69)	36(208)	
Unemployed	8(63)	6(13)	9(50)	
Homemaker/Student	8(58)	6(13)	8(45)	
Retired	39(301)	38(78)	39(223)	
Unable to work	9(79)	16(24)	8(45)	
Marital Status				<.001
Married/living w/ partner	57(443)	48(99)	60(344)	
Divorced/separated	18(135)	17(35)	18(100)	
Widowed	15(121)	20(41)	14(80)	
Never married	10 (46)	15(32)	8(46)	

*ANOVA (F-test) or χ2 tests to determine if differences exist based rural or urban residency.

**Table 2:** **Description including means, standard deviations and percent meeting recommendations for primary outcomes of fruit and vegetable (FV) intake, physical activity (PA) and BMI by residency**

Outcome	Total sample	Urban	Rural	p-value*
	(N=784)	(n=210)	(n=574)	
FV intake, M±SD cups/day	2.8±2.5	2.8±1.9	2.9±2.8	0.19
FV, % meeting recommendations	9	9	9	0.50
PA, Minutes of moderate-vigorous activity/week	127±182	122±182	132±183	0.49
PA, Minutes of strength training/week	25±205	18±77	31±256	0.65
PA, % meeting recommendations^a^	38	31	41	0.005
PA, % meeting recommendations^b^	11	9	12	0.33
BMI (kg/m^2^), M±SD	29.1±5.8	29.0±6.8	28.3±5.3	0.11
BMI, categorical %(n)^+^				
Normal Weight (18.05-24.9)	29(223)	32(66)	28(155)	<0.001
Overweight (25.0-29.9)	35(265)	24(48)	39(217)	
Obese (30–39.9)	31(231)	37(74)	29(157)	
Morbidly Obese (>40)	5(33)	7(14)	4(19)	

*p-value for either ANOVA (F-test) or χ^2^ tests to determine if differences exist based on urban or rural residency.

PA meeting recommendations^a^ = >150 minutes of moderate to vigorous activity.

PA meeting recommendations^b^= >150 minutes of moderate to vigorous activity plus 2 days of strength training activities.

^+^N=752, n=202 urban; n=550 rural. Differential responses due to missing data for BMI.

### FV intake

The average FV intake was 2.8(±2.5) cups per day, with only 9% of the sample meeting current recommendations The average cups of FV intakes and percentage of people meeting the recommendations were both low across all groups with no significant differences (F = 1.87, *p* = 0.67; F = 0.24, *p* = 0.50) based on rural (M = 2.9 ± 2.8) and urban (M = 2.8 ± 1.9).

### PA

The average minutes of moderate-to-vigorous physical activity (MVPA) per week was 127 ± 182 and there were no significant differences based on rural (M = 132 ± 183) or urban (M = 122 ± 182) classification (F = 0.47, *p* = 0.49). Thirty-eight percent of the total sample meets recommendations for MVPA and there were significant differences between rural and urban, with more rural residents reporting meeting recommendation for cardiovascular physical activity (χ^2^ = 6.90, *p* = 0.005). Only 11% meet the current full PA recommendations including both cardiovascular and strength training but these were not significantly different between rural and urban residents (F = 2.24, *p* = 0.33).

### BMI

The average BMI was 29.1 ± 5.8 and 40% of the sample was overweight, 29% obese and 3% morbidly obese. Examining continuous BMI, there are no significant differences between rural and urban participants (F = 2.51, *p* = 0.11). However, for categorical indicators of BMI, a higher percent of the rural sample was overweight and a higher percent of urban residents were obese or morbidly obese (χ^2^ = 18.38, *p* < .001).

### Prediction of health outcomes by rural or urban residency

Tables 3 and 4 illustrate regression results, accounting for individual characteristics, for continuous outcome models and dichotomous outcome models, respectively. Statistically significant (at 5% level) results are discussed here.

**Table 3:** **Linear regression model to test effects of residency on FV intake, physical activity and weight status when controlling for covariates**

Covariates	FV, Cups/day	PA, Min. of MVPA	BMI, kg/m ^2^
	β	β	β
Urban	-0.10	-4.53	0.55
Female	0.54**	-51.09**	-0.56
White	-0.08	22.00	-2.60***
College	0.54**	21.19	-0.51
Employed	-0.00	48.99**	0.33

*p<.05; **p<.01; ***p <.001.

**Table 4:** **Odds of residents meeting recommendations for FV intake, PA and weight status when controlling for covariates**

	FV, Meeting recommendations ^a^	PA, Meeting recommendations ^b^	BMI, overweight/obese ^c^
Covariates	OR (95% CI)	OR (95% CI)	OR (95% CI)
Urban	0.91 (0.52, 1.60)	0.65 (0.46, 0.93)*	0.78 (0.54, 1.12)
Female	2.25 (1.16, 4.34)*	0.63 (0.45, 0.88)**	0.74 (0.51, 1.08)
White	0.85 (0.49, 1.48)	1.19 (0.83, 1.71)	0.48 (0.31, 0.72)**
College degree	1.93 (1.15, 3.23)**	1.46 (1.07, 1.98)*	0.74 (0.79, 1.58)
Employed	0.85 (0.50, 1.43)	1.76 (1.29, 2.41)***	1.12 (0.79, 1.58)

*p<.05; **p<.01; ***p <.001.

^a^FV Meeting Recommendations= >5 cups of FV/day.

^b^PA meeting recommendations= >150 minutes of mod-vig activity plus 2 days of strength training activities.

^c^BMI is dichotomized to overweight/obese compared to normal weight.

There is no significant residency (i.e., urban) effect for FV intake or meeting FV recommendations (Tables 3 and 4). However, on average, females consume more FV than males (*p* < .05) and those with a college education (*p* < .01) consume more FV than those with lower education levels. This same relationship exists for models examining the probability of meeting FV recommendations in which females (OR = 2.14, *p* < .05) and college educated (OR = 1.74, *p* < .05) participants have increased odds of meeting FV recommendations (see Table 4).

Turning to PA models, while no significant differences in minutes of MVPA by residency (Table 3), urban residents are found to be less likely to meet PA recommendation (Table 4, OR = 0.65, *p* < .05) compared to their rural counterparts. An inverse relationship between gender and PA is shown, where on average females report fewer minutes of MVPA than males (Table 3) and the odds of meeting PA recommendation for males are 2.7 times more than the odds for females (*p* < .01; Table 4). Employed participants engage in more PA minutes per week than those who are unemployed (*p* < .01; Table 3). Employed participants (OR = 1.6, *p* < .001) and those with college degrees (OR = 1.35, *p* < .01) are more likely to meet recommendations for PA than unemployed or high school-educated counterparts (Table 4).

**Figure 1:**
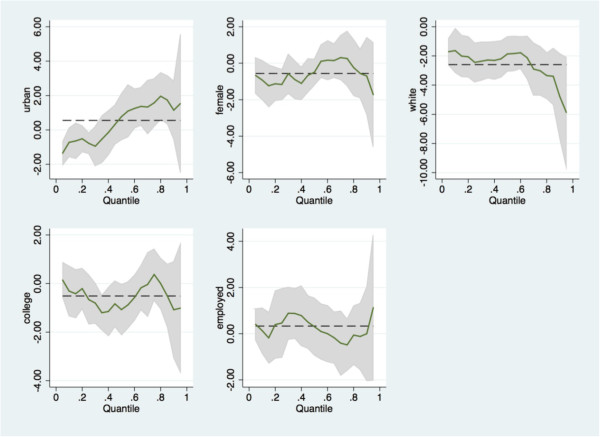
**Quantile regression models demonstrating effects of residency and demographic factors along the BMI distribution.** Note: The dependent variable is continuous BMI. The vertical axis shows the associated covariates while the horizontal axis shows the continuous BMI quantiles. The dashed lines denote the OLS regression coefficients estimates for the covariate shown in each panel; the solid lines denote the quantile regression coefficient estimates; the shaded areas are the 95% confidence intervals for the quantile estimates. Take the first panel for example: the dashed line shows the OLS estimates of the BMI differences between urban and rural (it shows that on average urban population is relatively heavier than rural but it is not statistically significant); the solid lines shows the quantile regression estimates of the BMI differences between urban and rural across the distribution of the BMI (it shows that the only statistically significant urban/rural gradient exists among those who had relatively smaller BMI).

We did not find a statistically significant relationship between residency and BMI, nor on gender, education status or employment status. However, on average, the white subgroup exhibits lower BMI levels compared to black (Table 3). When we consider BMI as a dichotomous outcome, combining overweight and obese to compare to normal weight, the race difference pattern is the same. For example, being white is protective against obesity, with 52-55% reduced odds of being overweight or obese for white residents compared to black residents (Table 4; OR = .45, *p* < .05 and OR = .48, *p* < .01). Further we tested the models stratified by race to explore differences between rural and urban residency and the dependent variables. There was not a significant effect for white/rural or white/urban on FV intake or BMI. However, for physical activity, urban whites were less likely (OR = .607, *p* < .05) to meet PA recommendations compared to rural whites. The models for black/rural and black/urban were not significant for any of the reported outcomes.We further examine potential heterogeneous effects of demographics and residency along the BMI continuous outcome distribution. We conducted quantile regressions to determine if residency gradients exist along certain segments of the BMI distributions that may differ from the residency average effects on BMI (Figure 1). Results show that positive and statistically significant gradients between urban and rural exist among the higher end of the BMI distribution (i.e., 80th percentile or higher). In other words, among those experiencing obesity, urban residents are even heavier than their rural counterparts (Figure 1). This shows that the severity of obesity is worse for those living in town limits compared to those living in rural area in this region. Another point worth noting is that there is a similar gradient based on race. Representation of white residents is higher at the lower ends of the BMI distribution, and there is a dramatic widening for blacks at the upper end of the BMI distribution. In other words, for those with obesity, the severity of obesity is worse among black compared to white (Figure 1).

## Discussion

These surveillance data provide a critical lens for identifying subgroups within the DRR that may benefit from programming targeting improvements in weight, FV, and PA outcomes. Additionally, comparing our findings to national and state level data helps the DRPHC to identify regional benchmarks and intervention targets.

In general, we demonstrate that the residents in the region fare worse on BMI compared to statewide estimates. The prevalence of obesity (BMI >30) at 36% is higher than statewide and national estimates of 29% and 28% respectively. Within our sample, the obesity prevalence among the urban population is 44% compared to 33% among the rural population [30].

Further, our results identify a disparity in BMI outcomes for those who are black and distinctions between urban and rural residents. Controlling for other covariates, blacks living in the region have higher BMIs than whites. The racial disparity is consistent with several other studies that show blacks and racial minorities have higher BMIs [3, 4, 7]. At the highest end of our BMI distribution, the gap between black and white residents widens demonstrating that more blacks are represented at the upper end. However, contradicting other national studies we find that rural residents have lower BMIs than urban counterparts, while controlling for sociodemographic factors [10, 12, 14]. Many of the studies examining rural and urban differences in health outcomes use national surveillance data such as NHANES [10, 14]. It may be that those findings are not applicable to the DRR or when considering a more nuanced designation for rurality such as the USDA RUCA codes. Given the high prevalence of obesity throughout the region compared to state and national averages a comprehensive approach including treatment and primary prevention will be necessary to reduce prevalence.

Important to addressing obesity is the promotion of healthful behaviors such as increased FV and PA. Regionally, intake of FV is very low. Only 9% of the population reported meeting recommendations compared to 27% of Virginians and 23% of Americans [30]. Our findings also indicate that participation in PA is low in the region with only 11% of the population meeting current recommendations that include cardiovascular and strength training compared to the 23% of Virginians and 29% in U.S. [34]. Due to differences in methodology and survey instruments, a precise comparison with state-level BRFSS PA is difficult. Nonetheless is it clear that few residents in this region met PA recommendation and that increasing participation in PA could help address obesity in the region.

Congruent with our hypothesis and other studies, we found that those with higher education are consuming more FV and more likely to be engaging in PA [12, 34–37]. We did not find these differences to be consistent by race. Although not an *a-priori* hypothesis, we find that women were consuming more FV but are less likely to meet PA recommendations which is supported by other studies [34–37]. In contrast to other studies and our hypothesis, we find that rural residents were more likely to be meeting PA recommendations [12, 38]. We did not support our hypothesis for a disparity in FV intake between rural and urban residents; however, the region-wide FV intake is very low. Efforts targeting increases in FV intake as part of an obesity prevention/reduction strategy could yield other health benefits to local residents. Further, regular PA confers a variety of health benefits that could improve other chronic health indicators (i.e. hypertension, diabetes) and reduce obesity.

### Strengths & limitations

This study is not without limitations. First, all telephone surveys, even with random sampling, have limitations for those who are likely to be contacted. Likewise, all survey data is fallible to self-report bias. We aimed to minimize these biases by including both land-lines and cell numbers and using previously validated survey instruments. Furthermore, these data represent a cross-sectional regional surveillance survey that provides a snapshot of the health indicators and may have limited generalizability outside the DRR. However, there is a clear need for local data, particularly for service partners working with these populations. The prevalence of health indicators on a local scale are important to identify target populations for programs, to promote decision making among local health and community organizations, and to allow for efficient use of resources.

## Conclusions

While obesity has been the primary focus of the DRPHC since its initiation in 2009 [23], these findings represent the first locally generated data to substantiate the region-wide magnitude of obesity-related health disparities within the DRR. The high prevalence of obesity, coupled with the low proportion of residents meeting FV and PA recommendations, clearly suggests that residents could benefit from primary prevention efforts and programming aimed at increasing positive health behaviors. Further, programming that targets those with BMIs >30 may be most effective in reducing obesity prevalence in the region. In particular, blacks, urban residents at the upper end of the BMI distribution appear to be at particular risk with prevalence in excess of their peers. Therefore, working with local partners from the DRPHC such as public health departments, public housing and parks and recreation to develop and implement programming that targets these hard to reach and vulnerable groups may be important next steps.

Finally, these data create an essential baseline for the ongoing work of the DRPHC. Measuring effectiveness is difficult for any coalition, and often effectiveness is measured by the success of a single program or intervention [39, 40]. Notably, this survey provides critical regional data that creates a benchmark to measure the success of obesity reduction efforts by the DRPHC over time. For example, since the time this survey was conducted, numerous research projects prioritized by the DRPHC have been launched, including objective assessments of the built environment [41, 42], a community-garden initiative [43], a pilot behavioral intervention targeting adult obesity [44], and a recently funded childhood obesity treatment planning grant [45]. Given the mission to reduce obesity, the DRPHC has the growing capacity to determine the potential public health impact of current and future obesity-related efforts in the region. Importantly, these surveillance data will be disseminated at the regional level through established partners and networks of the DRPHC, with the hopes of informing agenda setting and maximizing the development and implementation of high impact obesity reduction programs.
